# Supplementary material for: Aptamer-Based Biosensors for Rapid Detection and Early Warning of Food Contaminants: From Selection to Field Applications
Source: Molecules. 2025 Nov 7;30(22):4332. doi: 10.3390/molecules30224332 (PMC12655300; doi:10.3390/molecules30224332)
Supplement: Supplementary file 1 [file molecules-30-04332-s001.zip › molecules-3928515-supplementary.pdf]

Aptamer-Based Biosensors for Rapid Detection and Early Warning of Food Contaminants: From Selection to Field Applications

Table S1. Key configurations and performance parameters of different types of biosensors

Type	Structural design of sensor-bioreceptor complexes	Target	LOD	dynamic range	Time	Ref.
Electrochemical biosensors	Graphene/graphdiyne (GR/GDY) heterojunction-based sensing platform	Heavy metals	0.005 nM	0.003-5000 nM	-	[48]
Optical Biosensor	Integrated platform combining AgNPs@Cu-TCPP (Pt)/Au/TFBG sensors, optical channel arrays, and real-time signal processing modules	Heavy metals	0.007 (Pb^{2+}), 0.012 (Cd^{2+}) and 0.005 nM (Hg^{2+})	-	6 min	[56]
Optical Biosensor	Label-free photonic crystal aptasensor employing a SiO_2-Au-ssDNA 2D photonic crystal architecture (2D PC)	Antibiotics	1.10 pg/mL	5 pg/mL-5 μg/mL	45 min	[63]
Fluorescent sensor	Ratiometric fluorescent aptasensor utilizing AgNCs-SMP@ZIF-8 as the responsive signal and aptamer-functionalized CQDs as the reference	Antibiotics	-	0.98 nM	90 s	[64]
Electrochemical biosensors	FIS-based biosensor incorporating fully 2'-O-methylated RNA aptamers	Antibiotics	-	0.75-500 μM	5 min	[68]
Colorimetric biosensors	Paper-based colorimetric aptasensor	Antibiotics	300 nM		2 min	[70]
Fluorescent biosensor	Label-free fluorescent biosensor using specific aptamer-templated silver nanoclusters (AgNCs)	Antibiotics	11.46 ng/mL	20 ng/mL−10 g/mL	100 min	[76]
Electrochemical biosensors	Aptamer-functionalized graphene field-effect transistor (Apt-GFET) biosensor	Antibiotics	2.073 pM	-	500 s	[77]
Photoelectrochemical sensors	Novel self-powered anti-interference photoelectrochemical sensor via zirconium porphyrin-based metal–organic (ZPM) framework as multifunctional signal label	Antibiotics	0.03 pM	0.1 pM - 100 nM	-	[78]
Electrochemical biosensors	Versatile ampicillin aptasensor enabled by click chemistry on a graphene–alkyne derivative	Antibiotics	1.36 nM	-	-	[79]

Electrochemical biosensors	Label-free electrochemical biosensor utilizing a DNA tetrahedron-assisted aptamer	Antibiotics	0.69 nM	-	40 min	[80]
Electrochemical biosensors	Rapid test-strip integrating electrochemiluminescence with aptamer-gated mesoporous nanoparticles	Antibiotics	0.18 ± 0.07 μg l^{-1}	-	5 min	[81]
Electrochemical biosensors	Aptasensor was based on a dual-signal amplification strategy involving Nb2C-MB and Fe-N-C-CNTs	Antibiotics	0.094 nM(PenG), 0.093 nM (OXCL), 0.190 nM(AMO), 0.191 nM (AMP), and 0.125 nM()	-	40 min	[82]
Photoelectrochemical, electrochemiluminescence, and fluorescence biosensors	Multi-modal biosensing platform based on Ag-ZnIn2S4@Ag-Pt nanosignal probe-sensitized UiO-66	Antibiotics	3.48 fg/mL	-	200 s	[83]
Fluorescent sensor	Novel fluorescent probe leveraging Tb^{3+}-enrofloxacin aptamer coordination	Antibiotics	0.061 ng/mL (enrofloxacin), 0.020 ng/mL (norfloxacin), and 0.053 ng/mL(ciprofloxacin)	-	30 min	[89]
Biosensors	Multiplexed SELEX Yielding a Group-Specific DNA Aptamer for Biosensors	Antibiotics	0.14-0.71 μM	-	-	[90]
Photoelectrochemical aptasensor	Electrochemical aptasensor based on synergistically photosensitization enhanced by SYBR Green I and MoS2	Antibiotics	3.391 nM	20 - 1000 nM	-	[91]
Colorimetric sensor	Label-free biosensor constructed using DEX-specific aptamers and gold nanoparti-cle-modified graphene oxide	Hormonal molecules	-	20 - 100 nmol/ml	1 h	[95]
Electrochemical aptasensor	Molecularly imprinted electrochemical aptasensor based on dual recognition elements	Hormonal molecules	17.9 fM	10^{-13} - 10^{-5} M	30 min	[96]
Photoelectrochemical aptasensor	Self-power PEC aptasensor that used photoanode (FeOOH/In2S3) and photocathode (CuInS2) sim	Hormonal molecules	3.65 fg/mL	10 fg/mL - 1 μg/mL	1 h	[97]

	ultaneously to promote the generated cathodic p hotocurrent					
Electrochemical aptasensor	Electrochemical aptasensor for determination of t estosterone using an aptamer-nanogold-metal-orga nic framework-ionic liquid modified carbon past e electrode	Hormonal molecules	0.31 nM	1.0–50.0 nM and50.1 –1000.0 nM	-	[98]
Fluorescent aptasensor	Label-free fluorescent aptasensor based on G-qu adruplex structure	Hormonal molecules	0.41 Mm (EP) and 0.83 μ M (NP)	-	120 min	[100]
Electrochemical biosensor	Wearable electrochemical biosensor employing a CuMOF@InMOF-aptamer-gold nanoparticle co mposite	Hormonal molecules	0.27 nM	1 nM - 10 μM	1600 s	[101]
Fluorescent aptasensor	Aptasensor for the sensitive detection of EEs ba sed on luminescence resonance energy transfer	Hormonal molecules	0.022 ng/mL (DES) 0.045 ng/mL (HES)	0.065-64 ng/mL (DE S) and 0.0625 - 256 ng/mL (HES)	30 min	[102]
Electrochemical biosensor	Novel electro-chemiluminescence resonance ener gy transfer (ECL-RET) aptamer sensor based on NCDs@Ag3PO4 as a resonance energy transfer donor	Hormonal molecules	7. 4×10^{-14} M	1. 0×10^{-13} - 1. 0×10^{-6} M	30 min	[103]
Electrochemical aptasensor	Highly sensitive and specific electrochemical apt asensor modified by the BPs-aptamer was constr ucted	Hormonal molecules	6. 7 pM	-	-	[105]
Fluorescent aptasensor	Fluorescence aptasensing platform utilizing DNas e I–assisted cyclic enzymatic signal amplificatio n in conjunction with an aptamer/graphene oxid e complex	Hormonal molecules	0.3039 ng/mL (BPA), 2.643 ng/mL(E2), 0.6996 ng/mL (DES)	-	30 min	[106]
Colorimetric and electrochemical d ual-mode sensors	Colorimetric and electrochemical dual-mode sens ors assay for dual-mode detection of di-2-ethylh	Hormonal molecules	3.33×10^{-11}g/L	2.00×10^{-10}– 1.00×10^{-7}g·L^{-1} and 1.00	-	[107]

	exyl phthalate based on hemin-graphene nanocomposites			× 10^{-10}– 1.00 × 10^{-7} g·L^{-1}		
DNAzyme-based biosensors	Ultrasensitive detection platform for Staphylococcus aureus based on DNAzyme tandem blocking CRISPR/Cas12a	Bacteria	5 CFU/mL	-	29 min	[109]
Surface-enhanced Raman scattering biosensor	Surface-enhanced Raman scattering (SERS) biosensor based on gold nanostars (AuNSs)	Bacteria	1.0 CFU/mL	-	30 min	[110]
Surface-enhanced Raman scattering biosensor	Novel surface-enhanced Raman scattering (SERS) sandwich strategy biosensing plat-form	Bacteria	10 CFU/mL	-	55 min	[111]
Fluorescent aptasensor	FERT biosensor based on GO was designed for early detection of *A. baumannii* detection	Bacteria	1.1 CFU/mL	5 CFU/mL to 1 × 10^{5} CFU/mL	1.5 h	[112]
Electrochemical biosensor	Electrochemical aptasensor with N protein binding aptamer-complementary oligonucleotide as probe	Virus	-	10 fM - 100 nM	1 h	[114]
Electrochemical biosensor	Electrochemical aptasensor using screen-printed carbon electrodes	Virus	0.99 pM (R-21 electrode) and 1.11 pM(SD-2 electrode)	1 pM - 100 nM	30 min	[115]
Electrochemical biosensor	Combined aptamers with graphene field-effect transistors to develop a biosensor	Virus	6.17 pg mL^{-1}	-	5 min	[117]
Colorimetric aptasensor	Aptamer-mediated lateral flow assay (Apt-LFA) based on CuCo@PDA nanoenzymes	Mycotoxins	2.2 pg/mL	-	30 min	[129]
Colorimetric aptasensor	Colorimetric aptasensor for AFB1 detection by integrating Fe-N-C single-atom enzymes	Mycotoxins	1.5×10^{-7} ng/μL	-	2 h	[130]
Electrochemical aptasensor	Electrochemical aptasensor based on P-Ce-MOF @MWCNTs	Mycotoxins	1.0×10^{-5}ng/mL	5.0 × 10^{-5} - 50.0 ng/mL	2 h	[132]
Electrochemical aptasensor	Electrochemical aptasensor based on CRISPR/Cas12a-mediated and DNAzyme-assisted cascade dual-enzyme transformation strategy	Mycotoxins	6.27 × 10^{-6} ng·mL^{-1}	1×10^{-5} - 10 ng·mL^{-1}	1 h	[134]

Electrochemical aptasensor	Ultrasensitive aptasensor based the du-al-signal amplification capability of (Ce-In) Ox and COFT APB-DMTP@Au-Apt	Mycotoxins	7.6×10^{-8} ng mL^{-1}	5.0×10^{-7} ng mL^{-1} - 5.0 ng mL^{-1}	2 h	[136]
Electrochemical aptasensor	Aptasensor based on a target-induced strand displacement (TISD) strategy	Mycotoxins	8.74×10^{-7} ng/mL	-	2 h	[137]
Photoelectrochemical aptasensor	Self-powered photoelectrochemical aptasensor based on a Z-scheme $ZnIn_2S_4/WO_3$ photoanode	Mycotoxins	2.7 pg/mL	10 pg/mL–1000 ng/mL	16 s	[138]
Photoelectrochemical aptasensor	Self-powered photoelectrochemical aptasensor based on in situ topological conversion of Bi_2O_2S/Bi_2S_3	Mycotoxins	0.10 pg/mL	1 pg/mL - 100 µg/mL	1 min	[139]
Colorimetric aptasensor	Label-free colorimetric aptasensor using aptamer-enhanced oxidase-like activity of MnO2 nanoflowers	Mycotoxins	0.069 ng/mL	0.05 - 33.35 ng/mL	80 min	[141]
Colorimetric/electrochemical dual-mode aptasensor	Colorimetric/electrochemical dual-mode aptasensor with phosphatase-like ceria nanozyme	Mycotoxins	26 fg/mL (in electrochemical) and 10 pg/mL (in colorimetric mode)	100 fg/mL - 150 ng/mL	1 min	[143]
Optical biosensor	Aptameric photonic structure-based optical biosensor	Algal toxins	0.88 nM	3.8 nM - 150 nM	110 min	[146]
Electrochemical aptasensor	Electrochemical aptasensor using core-satellite gold nanoparticle/silver nanocluster nanoassemblies	Algal toxins	0.06 pM	0.2 pM - 200 nM	30 min	[147]
Colorimetric aptasensor	Aptamer-controlled reversible colorimetric assay based on highly active bimetallic organic frameworks	Algal toxins	0.07 ng/mL	0.1-200 ng/mL	-	[149]
Electrochemical biosensor	Electrochemical biosensor with aptamer/porous platinum nanoparticle on round-type micro-gap electrode	Algal toxins	4.669 pg/mL	10 pg/mL - 1 µg/mL	-	[150]
Fluorescent aptasensor	Aptamer microarray fluorescence detection based	Pesticide residues	25.4 ng/mL (phoxim), 12.0	-	30 s	[152]

	on thioflavin T		ng/mL (parathion), 7.7 ng/mL (fensulfothion), and 9.9 ng/mL (isocarbophos)			
Electrochemical biosensor	Dual-ratiometric aptasensor based on hairpin tetrahedral DNA nanostructures	Pesticide residues	-	4.3 pg /mL - 13.3 pg/mL	50 min	[153]
Electrochemical aptasensor	Ultra-sensitive electrochemical sensor based on PLL-BP and AuNPs	Pesticide residues	0.49 pM	1–10^5 pM	30 min	[154]
Electrochemical aptasensor	Highly selective electrochemical impedance spectroscopy-based aptasensor	Pesticide residues	1 nM	5 - 600 nM	12 h	[159]
Electrochemical aptasensor	'Apta-1' and 'Apta-2' aptamers were used to fabricate the gold electrode-based aptasensor platforms	Pesticide residues	-	range of ng/mL	30 min	[160]
Electrochemiluminescent (ECL) sensor	Electrochemiluminescence sensor based on Fe/Zn-BTC@C-dots sensitisation	Pesticide residues	9.81×10^{-12} mol/L	5-5000 × 10−11mol/L	10 min	[161]
Fluorescent aptasensor	Apta3-based fluorescent aptasensor	Pesticide residues	15.23 nmol/L	100 nmol/L-1500 nmol/L	15 min	[163]
Electrochemiluminescence aptamer sensor	Electrochemiluminescence aptasensor via ruthenium complex-modified dendrimers on multiwalled carbon nanotubes	Pesticide residues	9.6 pM	40 pM- 4 nM	30 min	[164]
Electrochemical aptasensor	Microfluidic chip containing a molecularly imprinted polymer and a DNA aptamer	Pesticide residues	67 pM	0.2 - 50 nM	5 min	[165]
Colorimetric aptasensor	Colorimetric aptasensor for λ-cyhalothrin	Pesticide residues	0.0186 μg/mL	-	3 min	[167]
Fluorescence resonance energy transfer aptasensor	Multicolor upconversion nanoparticles-black phosphorus nanosheet (UCNPs-BPNSs) biosensor	Pesticide residues	0.18 ng/mL(paraquat) and 0.45 ng/mL(carbendazim)	1.0 - 1.0 × 10^5 ng/mL	15 min	[168]
Visual aptamer-based chromatographic strip	Aptamer-based visual chromatographic strip (CS) technology using poly (dial-lyl-dimethylammonium chloride) (PDDA)	Pesticide residues	4.28 μg/L	20-150 μg/L	3 min	[169]

Fluorescent and ratiometric colorimetric biosensor	Innovative biosensing system integrating Fe-doped carbon dots (Fe-CDs) with Fe_3O_4@poly(dopamine) (PDA) nanoparticles	Preservatives	0.54 μM ($NaNO_2$) and 0.91 pM (KAN)	5-5000μM($NaNO_2$) and 17.2 pM−343 nM (KAN)	20 min	[176]
Colorimetric sensor	Colorimetric sensor constructed by combining Bor-A01, a high-affinity borax-specific aptamer obtained by SELEX technology screening, and gold nanoparticles (AuNPs)	Preservatives	0. 30-0. 50 μg/mL (in different food)	-	60 min	[177]
